# Dynamic contrast-enhanced magnetic resonance imaging and diffusion-weighted magnetic resonance imaging for predicting the response of locally advanced breast cancer to neoadjuvant therapy: a meta-analysis

**DOI:** 10.1117/1.JMI.5.1.011011

**Published:** 2017-11-24

**Authors:** John Virostko, Allison Hainline, Hakmook Kang, Lori R. Arlinghaus, Richard G. Abramson, Stephanie L. Barnes, Jeffrey D. Blume, Sarah Avery, Debra Patt, Boone Goodgame, Thomas E. Yankeelov, Anna G. Sorace

**Affiliations:** a University of Texas at Austin, Department of Diagnostics, Austin, Texas, United States; b University of Texas at Austin, Livestrong Cancer Institutes, Austin, Texas, United States; c Vanderbilt University, Department of Biostatistics, Nashville, Tennessee, United States; d Vanderbilt University, Center for Quantitative Sciences, Nashville, Tennessee, United States; e Vanderbilt University Medical Center, Department of Radiology and Radiological Sciences, Nashville, Tennessee, United States; f University of Texas at Austin, Institute for Computational and Engineering Sciences, Austin, Texas, United States; g University of Texas at Austin, Department of Biomedical Engineering, Austin, Texas, United States; h Austin Radiological Association, Austin, Texas, United States; I Texas Oncology, Austin, Texas, United States; j Seton Hospital, Austin, Texas, United States; k University of Texas at Austin, Department of Medicine, Austin, Texas, United States

**Keywords:** response prediction, dynamic contrast-enhanced MRI, diffusion-weighted MRI, breast cancer, neoadjuvant, meta-analysis

## Abstract

This meta-analysis assesses the prognostic value of quantitative dynamic contrast-enhanced magnetic resonance imaging (DCE-MRI) and diffusion-weighted MRI (DW-MRI) performed during neoadjuvant therapy (NAT) of locally advanced breast cancer. A systematic literature search was conducted to identify studies of quantitative DCE-MRI and DW-MRI performed during breast cancer NAT that report the sensitivity and specificity for predicting pathological complete response (pCR). Details of the study population and imaging parameters were extracted from each study for subsequent meta-analysis. Metaregression analysis, subgroup analysis, study heterogeneity, and publication bias were assessed. Across 10 studies that met the stringent inclusion criteria for this meta-analysis (out of 325 initially identified studies), we find that MRI had a pooled sensitivity of 0.91 [95% confidence interval (CI), 0.80 to 0.96] and specificity of 0.81(95% CI, 0.68 to 0.89) when adjusted for covariates. Quantitative DCE-MRI exhibits greater specificity for predicting pCR than semiquantitative DCE-MRI (p<0.001). Quantitative DCE-MRI and DW-MRI are able to predict, early in the course of NAT, the eventual response of breast tumors, with a high level of specificity and sensitivity. However, there is a high degree of heterogeneity in published studies highlighting the lack of standardization in the field.

## 1 Introduction

Neoadjuvant therapy (NAT) is widely considered the standard of care for the treatment of locally advanced breast cancer.^1^^,^^2^ NAT increases the success rate for breast conservation surgery by reducing tumor burden and provides the opportunity to treat micrometastases at an earlier time point compared to adjuvant treatment. Importantly, patients who achieve a pathological complete response (pCR; i.e., complete absence of viable tumor cells in the breast or axilla at the time of surgery) in the neoadjuvant setting have increased survival compared with patients who have residual disease at the conclusion of NAT.^3^^–^^7^ If it could be determined—early in the course of NAT—that a particular therapeutic regimen is unlikely to achieve a pCR, the treating physician could discontinue an ineffective treatment and substitute with an alternative regimen that may be more effective. With the numerous options for NAT that have become available, development of a method to predict response early in the course of therapy is especially needed.

The ability to predict which breast cancer patients will eventually achieve pCR presents a formidable challenge. Conventional, tissue-based biomarkers of response require a biopsy, which can include sampling errors due to tumor heterogeneity. Conversely, imaging approaches assess the entire tumor, obviating sampling error. However, imaging protocols require infusion of a gadolinium contrast agent, which may be retained in the brain,^8^ and must be optimized and validated for predicting tumor response to therapy. Furthermore, the optimal timing for image acquisition during NAT must be established to maximize the predictive ability of quantitative imaging.

The most commonly used method for quantitatively assessing the response of a tumor to NAT is the response evaluation criteria in solid tumors (RECIST).^9^ RECIST tracks regression in tumor size and has been shown to correlate with survival in a number of different cancers.^10^^–^^12^ In its current version, RECIST 1.1 uses imaging to identify, measure, and sum the longest dimension of up to five lesions prior to treatment. These dimensions are summed and compared with similar measurements post-treatment. The resulting change over time is categorized as complete response (disappearance of all target lesions), partial response (≥30% decline in sum of dimensions), progressive disease (≥20% increase in dimensions or appearance of new lesions), or stable disease (none of the preceding conditions met). However, there are a number of limitations in both making RECIST measurements accurately in the setting of irregular and/or indiscernible tumor margins, as well as in capturing tumor complexity with a single measurement. Furthermore, standard tumor size-based methods of evaluating response (including RECIST) lag behind a tumor’s biological response to treatment, such as cellular and vascular alterations.^13^^–^^15^ Moreover, size-based techniques may underestimate early efficacy for targeted agents exhibiting predominantly cytostatic rather than cytotoxic effects.^13^^,^^14^^,^^16^ Fortunately, a number of MRI techniques have matured to the point where they can offer a quantitative description of tumor characteristics that have shown the ability to predict the response of locally advanced breast cancer to NAT.^17^ In this meta-analysis, we focus on the two MRI methods that have accumulated the largest body of data for predicting the response of locally advanced breast cancer to NAT: dynamic contrast-enhanced MRI (DCE-MRI) and diffusion-weighted MRI (DW-MRI).

## 2 Rationale and Objectives

This meta-analysis assesses the ability of DCE- and DW-MRI for predicting, early in the course of NAT, which breast cancer patients will achieve pCR at the conclusion of NAT. Previous meta-analyses and systematic reviews evaluating the use of MRI in the neoadjuvant setting for breast cancer have not assessed its predictive value. A 2010 meta-analysis, performed by Yuan et al.,^18^ examined MRI performed following the completion of NAT. Similarly, a number of systematic reviews have investigated the ability of MRI to assess the response of breast cancer either immediately prior to and/or after the completion of NAT.^19^^–^^21^ This highlights a subtle, but important, distinction in that the present study focuses on MRI performed during the course of NAT, rather than the end of NAT, with the goal of predicting eventual response. A previous systematic review of MRI performed during NAT did not perform meta-analysis due to heterogeneity in MRI parameters and outcome definitions,^22^ factors that also limited inclusion in the present study.

For the purpose of this meta-analysis, we have focused on the techniques of DCE-MRI and DW-MRI for predicting response. We further subdivide DCE-MRI into semiquantitative measurements, which generate measures lacking direct physiological correlates, and quantitative measurements, which do have a direct physiological correlate. The intricacies of these techniques are discussed further below. We also include multiparametric studies combining measurements from both DCE-MRI and DW-MRI.

### 2.1 Semiquantitative Dynamic Contrast-Enhanced Magnetic Resonance Imaging

**Fig. 1:**
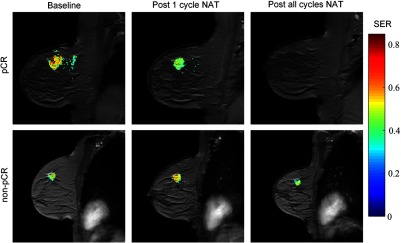
An example of semiquantitative DCE-MRI of a patient achieving pCR (top row) and a patient not achieving pCR (bottom row) before (first column), after the first cycle (second column), and at conclusion of all NAT (third column). The signal enhancement ratio (SER) overlaid on a high resolution anatomical MRI scan is shown. The high intensity regions-of-interest indicate gross tumor burden. The patient who ultimately achieved pCR demonstrates reduced tumor burden after one cycle of NAT.

DCE-MRI is an umbrella term used to describe a wide variety of dynamic MRI techniques and analytic approaches, including both qualitative and quantitative methods.^23^ Common to all approaches is the serial acquisition of heavily $T_{1}$-weighted images before and after the injection of the paramagnetic contrast agent. In the clinical setting, great emphasis is placed on obtaining DCE-MRI data at high spatial resolution,^24^ which necessitates a lower temporal resolution, resulting in time series data that can only be analyzed qualitatively or semiquantitatively. Semiquantitative analysis involves the extraction of descriptive parameters describing this time series data, such as enhancing volume over time, general curve shape features,^24^ or the ratio between the lesion intensity before and after contrast.^25^ Figure 1 displays an example of a semiquantitative analysis of DCE-MRI data obtained from a patient who achieved pCR (top row) and a patient who did not achieve pCR (bottom row) performed before, after the first cycle, and at the conclusion of all NAT. Application of semiquantitative DCE-MRI in the NAT setting has demonstrated that the percentage of tumor voxels demonstrating progressive (i.e., increasing signal intensity with time), plateau (steady intensity), or washout (decreasing intensity) kinetics predicts response to therapy.^26^ Alternately, comparison of early and late enhancement,^27^ measurements of peak signal enhancement,^28^ and changes in DCE-MRI time course shape^29^ are similarly predictive of pathological response. Importantly, the parameters derived from semiquantitative DCE-MRI provide predictive indicators of NAT response that are independent of traditional measures such as tumor size.^30^ The prognostic value of DCE-MRI was established in the I-SPY 1 trial, which showed that multivariate models incorporating semiquantitative DCE-MRI, histopathology, and breast cancer subtype were the most predictive of therapeutic response.^31^

### 2.2 Quantitative Dynamic Contrast-Enhanced Magnetic Resonance Imaging

**Fig. 2:**
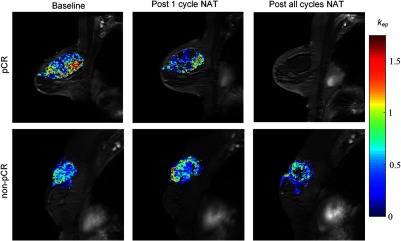
An example of quantitative analysis of DCE-MRI data of a patient achieving pCR (top row) and a patient not achieving pCR (bottom row) before (first column), after the first cycle (second column), and at conclusion of all NAT (third column). The parametric map of $k_{\mathrm{ep}}$ overlaid on a high resolution anatomical MRI scan is shown. Note the diminution of $k_{\mathrm{ep}}$ exhibited in the patient achieving pCR after one cycle of NAT, but the enhanced $k_{\mathrm{ep}}$ in the patient not achieving pCR.

In order to perform quantitative DCE-MRI, the following measurements are required: a precontrast $T_{1}$ map, high-temporal resolution dynamic $T_{1}$-weighted data, estimation of the time rate of change of the concentration of contrast agent in the blood plasma (i.e., the arterial input function, AIF), and a pharmacokinetic model to analyze the resulting data. By fitting the data to such a model (e.g., the Tofts model^32^), one can extract rate constants that reflect the influx of contrast agent into tissue ($K^{\mathrm{trans}}$, the volume transfer constant), efflux of contrast agent back into plasma ($k_{\mathrm{ep}}$), fractional volume of the blood plasma ($v_{p}$), and fractional volume of the extracellular extravascular space ($v_{e}$). This analysis can be conducted on a voxel-by-voxel basis allowing the construction of parametric maps. For example, Fig. 2 illustrates an example of $k_{\mathrm{ep}}$ map overlaid on anatomical images of a patient who achieved pCR (top row) and a patient who did not achieve pCR (bottom row) performed before, after the first cycle, and at the conclusion of all NAT. Following only one cycle of NAT, quantitative DCE-MRI parameters have been shown to be excellent predictors of pCR, whereas changes in longest dimension as characterized by RECIST were poor predictors of pCR even at the midpoint of NAT.^33^

### 2.3 Diffusion-Weighted Magnetic Resonance Imaging

**Fig. 3:**
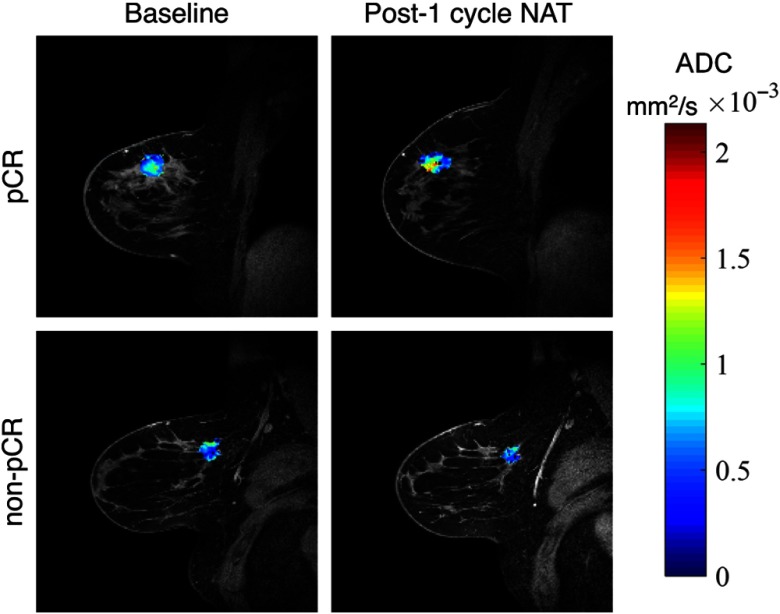
An example of DW-MRI data from a patient achieving pCR (top row) and a patient not achieving pCR (bottom row). ADC maps from the tumor region are overlaid on a high resolution anatomical image before (left column) and after the first cycle of NAT (right column). The patient who ultimately achieved pCR exhibits increased at ADC early time points within therapy, whereas the patient who did not achieve pCR does not.

The rate of water diffusion in cellular tissues can be described by an “apparent diffusion coefficient” (ADC), which depends, to a great extent, on the number and separation of barriers that a diffusing water molecule encounters. MRI methods have been developed to map the ADC and test–retest studies indicate that the ADC is highly repeatable and reproducible.^34^ Similar to DCE-MRI parametric mapping, the ADC can be computed on a voxel-by-voxel basis, yielding ADC parametric maps; for example, Fig. 3 presents representative ADC maps overlaid on anatomical images of a patient who achieved pCR (top row) and a patient who did not achieve pCR (bottom row) performed before and after the first cycle of NAT. As with DCE-MRI, DW-MRI has demonstrated the ability to predict response to therapy when performed during the course of NAT.^35^^,^^36^ Many publications have since appeared, validating the ability of DW-MRI to predict response in breast cancer. In particular, DW-MRI performed after the first cycle of NAT demonstrates that ADC increases in responders but not nonresponders and that changes in ADC correlate with decrease in tumor volume.^37^ Furthermore, changes in ADC may outperform measurements of tumor size for predicting tumor response to NAT.^38^ A multisite trial has confirmed that changes in ADC early during NAT are predictive of response with an area under the receiver operating curve of 0.825.^34^

### 2.4 Multiparametric DCE-MRI and DW-MRI

DCE-MRI and DW-MRI have separately demonstrated clear prognostic value in breast cancer NAT, but their greatest predictive value may result from combining the two techniques. Multiparametric methods that combine quantitative parameters from both DCE- and DW-MRI outperform metrics derived from either technique individually for predicting pCR either early in the course of NAT^39^ or following the completion of NAT.^40^ One study of integrated DCE- and DW-MRI indicated that $K^{\mathrm{trans}}$ and ADC are the most sensitive metrics to changes in the tumor occurring between initiation of NAT and surgery,^36^ although the optimal combination of multiparametric measurements is under investigation. Other studies have included results from not only DCE- and DW-MRI but also added magnetic resonance spectroscopy (MRS)^41^ or susceptibility-weighted MRI.^42^ We have included multiparametric studies comprising both DCE-MRI and DW-MRI in this meta-analysis in order to assess the benefit of combining DCE-MRI and DW-MRI measurements.

## 3 Methods

### 3.1 Identification of Eligible Studies

**Fig. 4:**
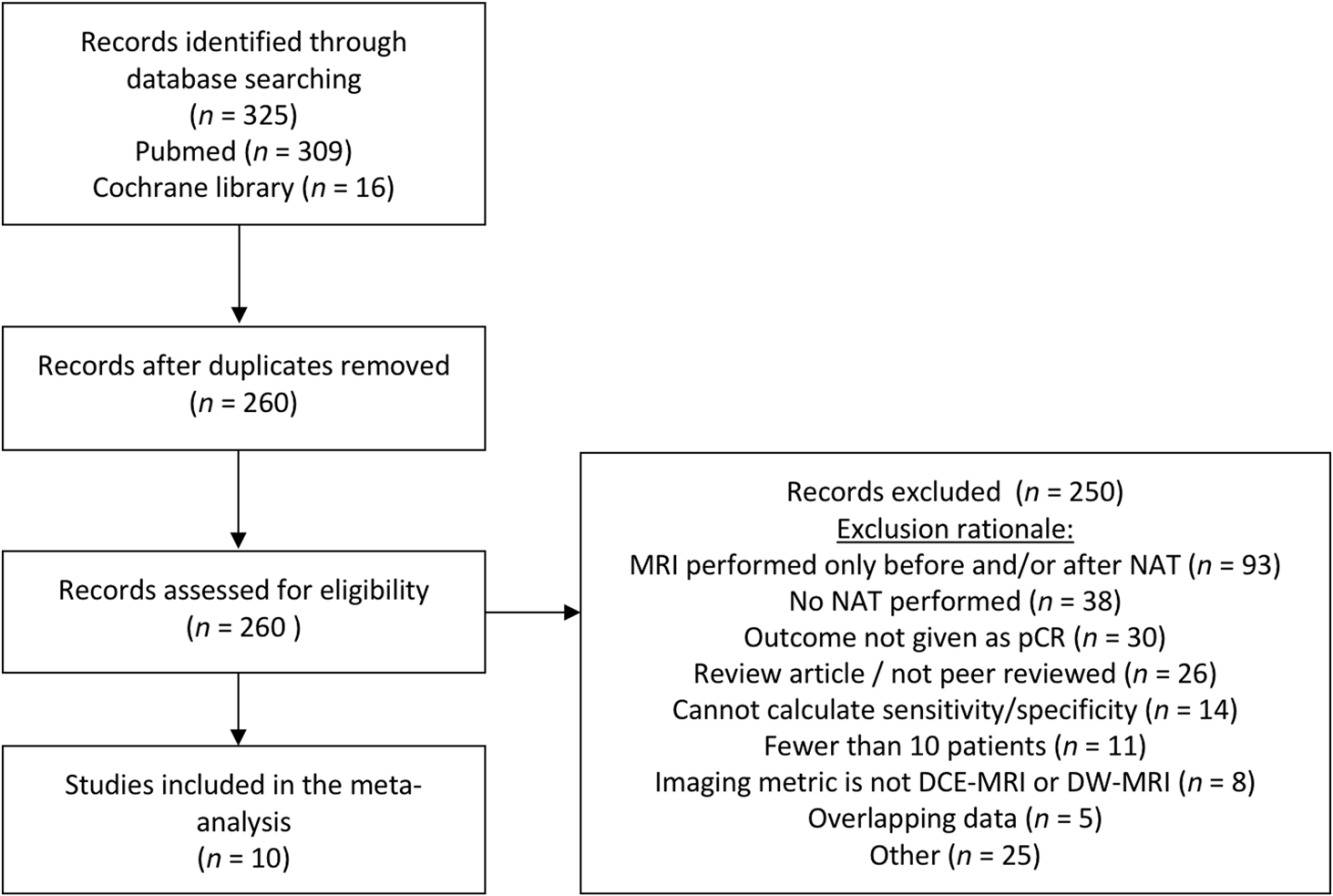
Overview of study selection. A total of 325 studies are identified from the Pubmed and Cochrane library databases. After removal of duplicates, 260 records were assessed for eligibility in this meta-analysis. After exclusion of 250 studies for reasons detailed in the flow chart, the remaining 10 studies were analyzed.

This meta-analysis was prospectively registered in the PROSPERO registry with the registration number CRD42016038770. The preferred reporting items for systematic reviews and meta-analyses checklist was followed when performing this meta-analysis.^43^ A comprehensive literature search was performed to identify studies reporting the sensitivity and specificity of DCE- and DW-MRI for predicting pCR in breast cancer patients receiving NAT as a component of their clinical care. The Pubmed and Cochrane library databases were searched from January 2001 through May 2017 using the following search terms: neoadjuvant and “breast cancer” and “contrast-enhanced” and MRI, preoperative and “breast cancer” and “contrast-enhanced” and MRI, neoadjuvant and “breast cancer” and diffusion and MRI, preoperative and “breast cancer” and diffusion and MRI. This search yielded a total of 325 studies eligible for meta-analysis. Duplicate studies were removed to yield 260 studies. From this list of 260 eligible studies, two reviewers (A.G.S. and J.V.) independently reviewed all studies according to the following inclusion criteria: must be reported in the English language, must report on human subjects, must include 10 or more subjects, peer-reviewed original article (no reviews, brief communications, or letters to the editor), sufficient data to determine specificity and sensitivity, the outcome measure must be pCR, MRI must be performed as a “predictive” measure (i.e., MRI must occur during the course of NAT, not after completion of NAT). Three studies met all inclusion criteria but included a receiver operative characteristic curve rather than providing sensitivity and specificity.^44^^–^^46^ For these studies, the corresponding author was contacted who then provided the sensitivity and specificity. Among reports with overlapping patient data, only the most recent publication was included in the meta-analysis. Studies were assessed for potential eligibility by first applying inclusion criteria on the abstract, followed by the full text if the abstract was deemed eligible or inconclusive. Data from eligible studies were extracted by two independent reviewers (A.G.S. and J.V.) with arbitration by a third reviewer (S.L.B.) in the case of disagreement. An overview of the study selection process is shown in Fig. 4. Ten studies met the stringent inclusion criteria of which three studies performed quantitative DCE-MRI, three studies performed semiquantitative DCE-MRI, two studies performed DWI-MRI, and two multiparametric studies reported MRI metrics, which combined results from both DCE-MRI and DWI-MRI.

### 3.2 Data Extraction

For each report, the following data were extracted into a standardized entry form: first author, journal, year of publication, number of cases, patient age (mean and range), initial clinical stage, histological subtype, receptor subtype [estrogen receptor positive (ER+), progesterone receptor positive (PR+), human epidermal growth factor receptor 2-positive (HER2+), triple negative], preoperative therapeutic regimen, MRI time points used for prediction, pCR rate, magnetic field strength, contrast agent, contrast agent dose, DCE-MRI temporal resolution, b-values for DW-MRI acquisition, and either sensitivity/specificity or sufficient data to construct a 2×2 contingency table. Additionally, the MRI type was categorized as either semiquantitative DCE-MRI (in which the MRI parameter does not have direct physiological units), quantitative DCE-MRI (MRI parameter has physiological units), DW-MRI, or multiparametric (combination of DCE-MRI and DW-MRI). The MRI parameter used to define the sensitivity and specificity was also extracted. A summary of these parameters for studies included in this meta-analysis is given in Table 1.

**Table 1:** Description of studies included in the meta-analysis.

Study	Year	No. of patients	Patient age, mean (range)	Initial clinical stage	Histological subtype	Receptor status	Preoperative therapy regimen	Time between start of NAT and MRI	pCr rate	MRI magnetic field strength	Contrast agent	DCE temporal resolution (s)	DW-MRI b values	Sensitivity	Specificity
Semiquantitative DCE-MRI studies															
Parikh^47^	2014	36	49.8 (24–67)	I–III	35 IDC, 1 ILC	21 ER+, 6 HER2+	3*FEC → 4*T, 4*EC → 4*T HER2+ patients after 2010 received trastuzumab	3 cycles NAT	22.22%	1.5T	Dotarem (20 mL)	92		87.5%	82.1%
Rigter^48^	2013	246	48 (18–68)	I–IV	190 IDC, 33 ILC, 6 IDC/ILC	190 ER+, 246 PR+	3*AC → 3*AC, 3*AC → 3*TCa	3 cycles NAT	1.63%	1.5T	Gadolinium CA (0.1 mmol/kg)	90		75.0%	33.2%
Yang^46^	2012	22	45 (35–67)	II–III	Not reported	Not reported	Antiangiogenic-cytotoxic combination therapy	2 cycles NAT	27.27%	1.5T	Magnevist (0.1 mmol/kg)	70		78.0%	100.0%
Quantitative DCE-MRI studies															
Tateishi^49^	2012	143	57 (43–72)	I–III	131 IDC, 11 ILC	100 ER+, 82 PR+, 111 HER2+	4*FEC → 12*T, 4*AC → 12*T, HER2+ patients received trastuzumab	2 cycles NAT	16.90%	3T	Magnevist (0.1 mmol/kg)	10		51.7%	92.0%
Ah-See^50^	2008	28	46 (29–70)	II–III	21 IDC, 3 ILC	Not reported	6*FEC	2 cycles NAT	39.28%	1.5T	Magnevist (0.1 mmol/kg)	12		94.0%	82.0%
DWI-MRI studies															
Fangberget^51^	2011	31	50.7 (37–72)	Not reported	24 IDC, 7 ILC	21 ER+, 18 PR+, 11 HER2+, 5 TNBC	4*FEC → 2*FEC, 4*FEC-> 2*T, HER2+ patients received trastuzumab	4 cycles NAT	35.48%	1.5T			100, 250, 800	88.0%	80.0%
Minarikova^44^	2017	42	52 (29–74)	I–III	41 ID, 1 ILC	27 ER+, 13 PR+, 5 HER2+	ACT*6, ACT*8, AC → T	2 cycles NAT	16.67%	3.0T			0, 850	66.67%	100%
Multiparametric MRI studies															
Li^39^	2015	37	45 (28–67)	II–III	Not reported	16 ER+, 16 PR+, 12 HER2+, 12 TNBC	4*AC → 4*T, 4*AC → 12*T, HER2+ patients received trastuzumab or other treatments	1 cycle NAT	35.14%	3T	Magnevist (0.1 mmol/kg)	16	0, 500	92.0%	75.0%
Wu^45^	2015	31	48.4 (33–62)	II–III	Not Reported	24 ER+, 23 PR+, 16 HER2+	FEC, FEC → T, T, or other treatments	1 cycle NAT	9.70%	3.0T	Gadovist (0.1 mmol/kg)	40–50	50, 600, 1000	90.9%	83.8%

Note: IDC, invasive ductal carcinoma; ILC, invasive lobular carcinoma; TNBC, triple negative breast cancer; A, doxorubicin; C, cyclophosphamide; T, taxane; F, fluorouracil; E, epirubicin; D, docetaxel; Ca = capecitabine.

The methodological quality of each study in this meta-analysis was assessed using the quality assessment of diagnostic accuracy studies (QUADAS) tool.^52^ Using the QUADAS tool, an overall quality score was calculated (maximum 14). The average number of patients in the studies in the meta-analysis was 64, and the minimum number of patients was 22.

### 3.3 Statistical Analysis

All analyses were performed using Stata version 14.1 (StataCorp, College Station, Texas) and R version 3.3.3 (R Foundation for Statistical Computing, Vienna, Austria). The sensitivity, specificity, and log diagnostic odds ratios were calculated along with the associated 95% confidence intervals and overall adjusted estimates were pooled across all studies. The amount of heterogeneity across the studies was evaluated via the $I^{2}$ statistic, which represents the percentage of total variation in meta-analysis that can be attributed to between-study heterogeneity. This heterogeneity was taken into account in the evaluation of test performance with a hierarchical summary receiver operator curve (HSROC).^53^ The estimation of the HSROC uses a Bayesian hierarchical approach to account for both within- and between-study heterogeneity. Publication bias was examined graphically via a funnel plot in which the diagnostic odds ratio is plotted versus the inverse root of the effective sample size and was tested using Deek’s method. Also, note that any empty cells in the 2×2 table setup were replaced with 0.1 to align with requirements of statistical programming software as well as to allow for calculation of diagnostic odds ratios.

The large degree of heterogeneity in this meta-analysis implies that the data should not be pooled in a fixed-effect model. As a result, a random effects logistic regression model was fit to model sensitivity and specificity while accounting for differences between individual studies that are not due to study characteristics. The meta-regression analysis is given by the following equation:

$$\mathrm{logit}[\mathrm{Pr}({\mathrm{MRI}}_{ij}=1|X_{ij})]=(\beta_{0}+b_{0i})+\beta_{1}\cdot {\mathrm{pCR}}_{ij}+\beta_{2}\cdot {\mathrm{ER}}_{i}+\beta_{3}\cdot {\mathrm{PR}}_{i}+\beta_{4}\cdot \mathrm{HER}2_{i}+\beta_{5}\cdot {\text{Age}}_{i}+\beta_{6}\cdot {\text{Temporal Resolution}}_{i}+\beta_{7}\cdot \mathrm{MRI}\;\text{Type}+\beta_{8}\cdot \mathrm{NAT}\;\text{cycle}+\beta_{9}\cdot {\mathrm{pCR}}_{ij}\cdot \mathrm{HER}2_{i}+\beta_{10}\cdot {\mathrm{pCR}}_{ij}\cdot {\text{Age}}_{i},$$

where i denotes the study (i=1,…,10) and j denotes the individual within study i. ${\mathrm{MRI}}_{ij}$ represents the MRI prediction of pCR for the j’th patient within study i; ${\mathrm{MRI}}_{ij}=1$ represents an MRI prediction of pCR; and ${\mathrm{MRI}}_{ij}=0$ represents an MRI prediction of a non-pCR (i.e., residual tumor remaining at time of surgery after NAT). Similarly, a patient with ${\mathrm{pCR}}_{ij}=1$ achieved a pCR and ${\mathrm{pCR}}_{ij}=0$ indicates non-pCR. ER, PR, and HER2 are percentages of each study population that exhibited overexpression of each receptor, respectively. Age is mean age within each study. Temporal resolution and the number of NAT cycles (NATcycle) between start of NAT and MRI were constant within each study. MRI type was constant within each study and took values of either quantitative DCE, semiquantitative DCE, multiparametric, or DWI. Note that the inclusion of ${\mathrm{pCR}}_{ij}$ in the model in combination with the outcome of ${\mathrm{MRI}}_{ij}$ allows for our calculation of sensitivity, specificity, and diagnostic odds ratio.

To fit the regression model, an expanded dataset was generated, as previously described.^54^ In the expanded dataset, each study was given a row for each study subject, where each subject was classified as true positive, false positive, false negative, or true negative. Study-level covariates remained constant for all subjects while MRI test result (i.e., positive or negative) and pCR status were pseudoindividual specific. This expanded dataset allows for analysis to be conducted at the pseudoindividual level. Individual study characteristics included in the regression included: percent of the study population that were ER+, percent of the study population that were PR+, percent of the study population that were HER2+, mean age, number of NAT cycles between start of NAT and MRI, DCE temporal resolution, and magnetic field strength (1.5T or 3T). MRI type was highly correlated with magnetic field strength (1.5T or 3T) and magnetic field strength was thus dropped from the final model. Interactions between each variable and pCR status were considered and included if deemed of scientific interest; i.e., the effect of HER2 status on the association between MRI test result and pCR status. We also assessed interactions between pCR and the other hormone status, but as these were not statistically significant, they were not included in our model. The interaction terms were assessed for their effects on both sensitivity and specificity by likelihood ratio tests while adjusting for the effects of other variables in the model. Not all studies reported temporal resolution, ER, PR, and HER2 rates for their individual study populations, thus, multiple imputation with chained equations was utilized to allow for the inclusion of these studies without loss of information that would result from a complete-case analysis.^55^ In this process, 10 imputed datasets were created, where pCR rate for each study was used to predict the temporal resolution, ER, PR, and HER2 rates that were missing in the original data. These imputed datasets are used to calculate parameter values and variances for temporal resolution, ER, PR, and HER2 in the metaregression analysis.

The logistic regression model provides adjusted estimates of sensitivity and specificity for each study that can be averaged to calculate average adjusted estimates for both sensitivity and specificity. Subgroup analysis was performed according to the type of MRI performed (semiquantitative versus quantitative DCE-MRI). In this process, information was pooled across semiquantitative studies and quantitative studies, respectively, resulting in two independent 2×2 tables. A simple test of the difference between two independent means was conducted for each of the three outcomes of interest: log diagnostic odds ratio, sensitivity, and specificity.

## 4 Results

### 4.1 Meta-analysis Outcome Measures

The funnel plot of diagnostic odds ratio versus the inverse root of the effective sample size revealed indicated a lack of asymmetry and, consequently, no evidence of publication bias (p=0.34). The selection of only 10 studies reveals possible publication bias, though the small sample size prevents a clear conclusion. The high $I^{2}$ statistic calculated in this meta-analysis (90%) suggests a high degree of heterogeneity present among studies. Additionally, the unadjusted pooled analysis revealed a significant degree of heterogeneity among the 10 included studies in both sensitivity (78.8%) and specificity (99.5%). Pooled, unadjusted estimates of sensitivity and specificity for MRI in predicting pCR are given in Table 2. Due to the high amount of heterogeneity, we chose to fit a random effects metaregression model to help account for differences between individual studies.

**Table 2:** Pooled sensitivity, specificity, and diagnostic odds ratios for the 10 studies in this meta-analysis. The unadjusted values were calculated without adjusting for covariates, while the adjusted values were adjusted for covariates according to the logistic regression model. Both sets of values take into account possible heterogeneity between studies via a random effects model. These values, along with the individual sensitivities, specificities, and diagnostic odds ratios for individual studies, are displayed visually in the forest plots in Fig. 6.

	Unadjusted-mean (95% CI)	Adjusted-mean (95% CI)
Sensitivity	0.85 (0.70, 0.93)	0.91 (0.80, 0.96)
Specificity	0.86 (0.72, 0.94)	0.81 (0.68, 0.89)
Diagnostic odds ratio	35 (11, 107)	42.81 (13.67, 134.06)

**Fig. 5:**
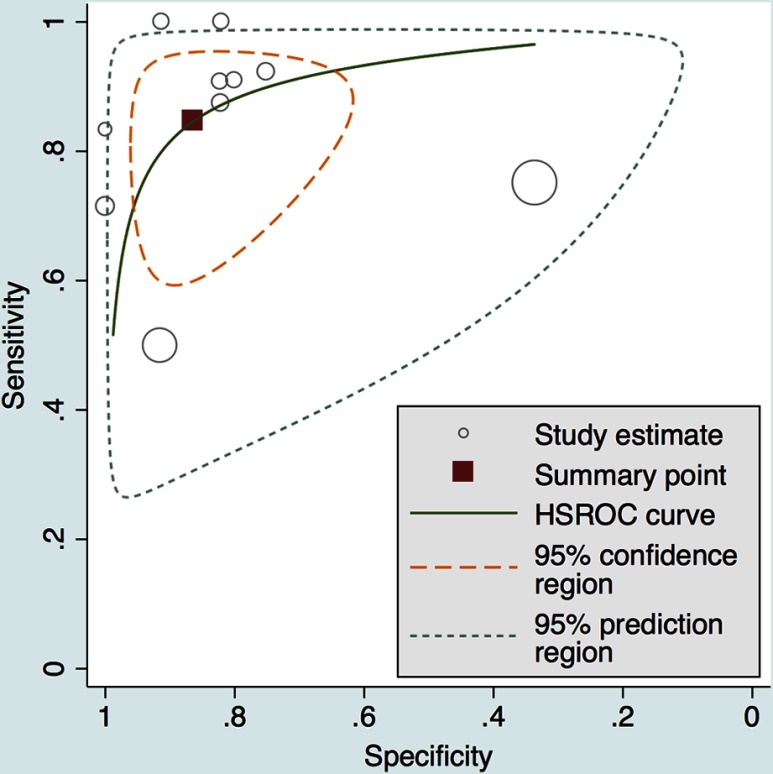
The HSROC curve displays test performance of individual studies as well as the pooled average estimate. The summary operating point and large area under the curve demonstrate that DCE- and DW-MRI can achieve high sensitivity and specificity for predicting pCR in the neoadjuvant setting for breast cancer. The width of the confidence contour demonstrates the high amount of heterogeneity present in the included selection of studies. Each circle represents a study and the size of the circle refers to the size of the study.

Test performance of the MRI methodologies across all studies is summarized in the HSROC, where individual studies are shown alongside the pooled estimate (Fig. 5). This figure was created via the hierarchical model from Rutter and Gatsonis.^53^ The area under the HSROC curve was 0.92 (95% CI, 0.89–0.94). The width of the 95% confidence contour demonstrates the amount of heterogeneity present between the 10 studies. Of note, 4 of the 10 studies cluster closely to this receiver operating curve. The two largest studies are seen to have considerable influence over the width of the prediction contour. The width of the prediction contour demonstrates the uncertainty with which we may predict the results of a future study. The variation among studies included in this meta-analysis does not allow for a targeted prediction of the performance of an unobserved future study.

The studies had QUADAS scores ranging from 10 to 12. Due to the nature of the MRI being performed early in all studies, the time between the MRI and pathological evaluation was not short enough to be reasonably sure that the target condition did not change between MRI and pathology. Furthermore, it was not clear that the MRI result was evaluated without knowledge of the pathology results. Some studies did not explain study withdrawals or uninterpretable test results sufficiently.

### 4.2 Metaregression Analysis

**Fig. 6:**
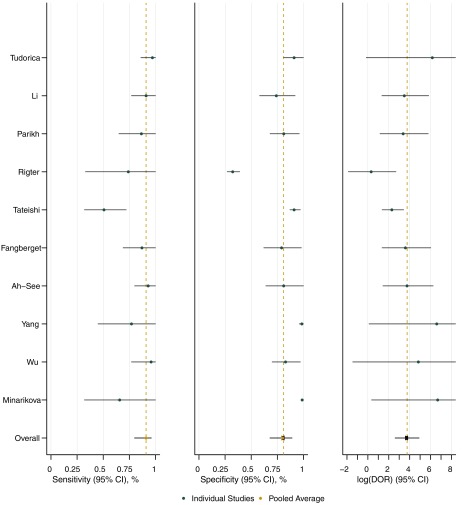
Individual study estimates for sensitivity, specificity, and log diagnostic odds ratio are displayed in the forest plot along with the overall average adjusted estimates (from the random effects model) and 95% confidence intervals for each. The pooled average demonstrates that DCE- and DW-MRI have high sensitivity, specificity, and log diagnostic odds ratio for predicting response to NAT in breast cancer.

The random effects model described in Sec. 3.3 was fit to account for possible heterogeneity between studies. In this technique, each study has allowed its own unique baseline effect while the covariate effects were assumed to be similar across studies. This regression model allows for estimates of both sensitivity and specificity to be adjusted for study-specific covariate values. These adjusted estimates are shown in Table 2 along with similar estimates that have not been adjusted for covariates. Note that adjusting for covariates resulted in a small decrease in estimated specificity (0.81 adjusted versus 0.86 unadjusted), whereas the sensitivity (0.91 adjusted versus 0.85 unadjusted) and diagnostic odds ratio (42.81 adjusted versus 35 unadjusted) were increased by adjustment for covariates. A forest plot of individual study estimates of sensitivity, specificity, and log diagnostic odds as well as overall average adjusted estimates is given by Fig. 6. The forest plot visual demonstrates the heterogeneity among studies as well as the variation that is present within each individual study.

Using our random effects model, we found that both MRI type and the number of cycles of NAT before the MRI had significant effects on the sensitivity and specificity simultaneously, at a 95% confidence level. MRI performed earlier after the start of NAT (fewer NAT cycles prior to MRI) led to increased specificity but decreased sensitivity compared with MRI performed later in the course of treatment. For both analyses, the models took into account the effect of other covariates in the model; i.e., ER, PR, temporal resolution, and magnetic field strength. The parameter estimates as well as standard errors and p-values for the metaregression analysis are shown in Table 3.

**Table 3:** The parameter estimates as well as standard errors and p-values for the meta-regression analysis are shown. In this analysis, MRI type and MRI time were both statistically significant at a 95% confidence level. Note that, while pCR has a statistically significant coefficient, this parameter is used in the calculation of sensitivity, specificity, and diagnostic odds ratio, rather than having an interpretation of its own.

	Coefficient	Standard error	p-value
Log (ER)	−1.25	17.75	0.945
Log (PR)	0.97	7.53	0.899
Log (HER2)	1.61	9.27	0.863
pCR	8.90	4.40	0.043
pCR*log (HER2)	−1.96	2.04	0.336
Mean (age)	−0.12	0.08	0.130
pCR*mean (age)	−0.09	0.10	0.375
Temporal resolution	0.02	0.08	0.757
MRI type	1.59	0.56	0.005
NAT cycle	1.65	0.54	0.002
Intercept	−3.12	4.29	0.467

### 4.3 Subgroup Analysis

A subgroup analysis was performed to determine if semiquantitative and quantitative DCE-MRI were statistically different in terms of sensitivity, specificity, and diagnostic odds ratios. Three quantitative DCE-MRI (199 total subjects) and three semiquantitative DCE-MRI (304 total subjects) studies were included in this subgroup analysis. T-test analysis indicates that quantitative techniques resulted in higher specificity and log diagnostic odds ratio (p<0.001 and p=0.014, respectively) at a 5% significance level. Semiquantitative techniques resulted in higher sensitivity than quantitative techniques, though this difference is not statistically significant at a 5% level (p=0.13).

## 5 Discussion

This meta-analysis identified 10 studies performing DCE- or DW-MRI during the course of NAT for breast cancer that reported the sensitivity and specificity for predicting pathological response. The pooled sensitivity, specificity, and diagnostic odds demonstrate the potential of MRI for early prediction of response to NAT. However, there was a high degree of heterogeneity between these studies, as expected, given the variation in MRI methods and patient populations. A random effects model examining differences between studies in terms of patient population (patient age, percent of ER, PR, and HER2+) and imaging protocol (magnetic field strength, temporal resolution of DCE-MRI, cycle of NAT) found that the type of MRI performed and the time between the start of NAT and MRI significantly influence sensitivity and specificity simultaneously. MRI performed earlier after the start of NAT had increased specificity but decreased sensitivity for predicting pCR. This highlights the need to identify the best time point for predicting response. While this study may be underpowered to detect the influence of other covariates due to the small number of studies, it suggests that accuracy of prediction of response is not influenced by differences in patient populations or other imaging parameters included in the random effects model.

Although this study sought to compare quantitative DCE-MRI, semiquantitative DCE-MRI, DW-MRI, and multiparametric approaches incorporating both DCE- and DW-MRI, the limited number of studies reduced our ability to compare these groups. There were only two multiparametric studies and two DW-MRI studies, which met the inclusion criteria for this meta-analysis. Subgroup analysis comparing quantitative and semiquantitative DCE-MRI indicated that quantitative techniques had a higher specificity and log diagnostic odds ratio. Further studies are needed to validate whether the added rigor of quantitative DCE-MRI translates into improved predictive power for MRI in the neoadjuvant setting. Studies comparing quantitative and semiquantitative DCE-MRI performed on the same data would be especially useful for this evaluation.

The number of studies included in this meta-analysis was limited by heterogeneity in the reported metrics on patient outcome. The success of NAT for breast cancer is commonly measured either in terms of patient survival or pathological response at resection. We confined this meta-analysis to studies reporting patient outcome as pCR, indicating a lack of positive tumor margins or nodal involvement at the time of resection. However, our study design excluded a number of excellent studies, which reported NAT success in terms of patient survival.^30^^,^^31^^,^^56^^–^^60^ Survival and pCR correlate, as demonstrated by pooled analysis of nearly 12,000 women with breast cancer; however, this study noted heterogeneity in this relationship as a function of receptor subtype.^61^ The correlation between recurrence free survival and pCR is demonstrably improved when stratified by receptor subtype.^62^ Given this dependence on receptor subtype, we were unable to combine studies reporting pCR and survival in this meta-analysis. Even among studies reporting outcome in terms of survival, some outcomes were reported as overall survival whereas others were given as disease-free survival. Similarly, studies reporting pathological response commonly grouped complete and partial responses.^34^^,^^63^ The lack of standardization in outcome metrics confounds meta-analyses and hampers direct comparison of different imaging techniques.

Lack of standardization is similarly of concern in both image acquisition and processing. There are a number of parameters that can be adjusted in MRI acquisition, and there is currently no consensus on the optimal settings for acquiring images. To the best of our knowledge, there are no multisite, multivendor studies validating the quantitative MRI techniques currently in use.^64^ The quantitative imaging network (QIN) is attempting to address this shortcoming by standardizing methods for image acquisition, analysis, and sharing.^65^ Additionally, the QIN seeks to cross-calibrate imaging results obtained at different centers and build reference datasets for development and validation of new quantitative imaging methods. There is also a paucity of clinical results assessing the repeatability through test–retest studies and reproducibility, through multisite trials, of quantitative MRI metrics performed in the breast.^66^^,^^67^ In order to be adopted as routine biomarkers, the repeatability of quantitative MRI metrics must be established and validated with multisite studies.

Image processing of quantitative and semiquantitative MRI is an area of active research. One burgeoning field, known as radiomics, uses computer-aided image processing to extract a large number of quantitative parameters from each image. Radiomics has recently been applied to imaging in breast cancer NAT. Indeed, one of the studies included in this meta-analysis employs a radiomic approach to describe the irregularity of contrast-enhanced MRI.^47^ Texture analysis of breast cancer DCE-MRI demonstrates differences between responders and nonresponders.^68^ Radiomic analysis can be performed on both quantitative and semiquantitative DCE-MRI and comparisons of these approaches are needed. There are also a number of predictive models under development that integrate multiple parameters extracted from quantitative MRI. For example, mathematical models based on tissue mechanical properties and constrained by DW-MRI data may be useful for predicting pCR.^69^ Alternately, predictive models incorporating DCE- and DW-MRI to estimate tumor cell proliferation can be used to predict pCR.^70^

There are a number of other imaging techniques being applied to image the breast, which were not included in this meta-analysis. One such technique is the use of MRS to characterize the concentration of certain tumor metabolites. For instance, MRS can detect choline, a marker of high cellular turnover, which is elevated in certain tumors and inflammatory processes. MRS following NAT demonstrates a decline in choline concentration, although this may not predict pCR as well as DW-MRI.^71^ Alternately, MRS has been used to measure the ratio of water signal to fat signal early in the course of NAT and found that this ratio may have predictive power.^72^ Positron emission tomography (PET) is another promising method for detecting molecular changes in tumors in response to therapy. A multimodal study combining DCE-MRI and fluorodeoxyglucose (FDG)-PET found that both techniques were able to independently predict disease free survival, but that the combination of MRI and PET gave the best prognostic value.^57^ A similar result was found when combining DW-MRI and FDG-PET after the completion of NAT, wherein the combination of MRI and PET metrics improved the specificity of predicting response.^73^ The fusion of quantitative multimodal imaging techniques, such as MRI and PET, can provide a more complete characterization of the tumor and provide a voxel-level fusion of complementary imaging data^74^ that can be coregistered longitudinally over the course of NAT.^75^ A third imaging technique, currently in its early stages, is the application of near-infrared light to perform diffuse optical tomography of the breast.^76^ A pilot study of diffuse optical tomography demonstrates that the technique can detect vascular alterations in breast cancer early in the course of NAT.^77^ A direct comparison between DCE-MRI and diffuse optical tomography demonstrated that optical tomography differentiates responders and nonresponders as early as the first cycle of treatment and is equally effective in predicting response as DCE-MRI.^78^

## 6 Conclusion

This comprehensive meta-analysis demonstrates that DCE- and DW-MRI performed during breast cancer NAT can predict pathological response across a range of studies, an exciting and important finding with potential clinical implications. However, the present study also highlights a high degree of heterogeneity (both in patient population and image acquisition/analysis) in the field. Moreover, it reveals a lack of studies that have simultaneously investigated DCE-MRI and DW-MRI as predictive methods to evaluate response to treatment early in the course of NAT. Further development and evaluation of new and established MRI techniques in breast cancer NAT would benefit from standardization in study design, patient population, and reported metrics of prediction accuracy. Additionally, the type of MRI analysis performed may also influence predictive accuracy, as suggested by the improved specificity of quantitative dynamic contrast-enhanced versus semiquantitative techniques.
